# Dynamic Constitutive Relationship of Mg–Gd–Y–Zr–Ag Alloy during High Temperature Deformation Process

**DOI:** 10.3390/ma16072587

**Published:** 2023-03-24

**Authors:** Shunli Peng, Yunxin Wu, Tao Zhang, Qiumin Xie, Zhongyu Yuan, Lan Yin

**Affiliations:** 1 Light Alloy Research Institute, Central South University, Changsha 410083, China; 2 State Key Laboratory of High-Performance Complex Manufacturing, Central South University, Changsha 410083, China; 3 School of Mechanical and Electrical Engineering, Central South University, Changsha 410083, China

**Keywords:** deformed magnesium alloy, flow stress behavior, Johnson-Cook model, hot deformation

## Abstract

The thermal deformation behavior of the Mg–Gd–Y–Zr–Ag alloy was studied by isothermal hot compression tests at high temperatures. The flow stress increased with increased strain rates and decreased temperatures, first increasing and finally remaining stable with increased strain. A hot processing map was built. Using the processing map and microstructural analysis, the temperature should remain at 673–773 K for this alloy to ensure the deformation quality. The primary softening mechanism is discontinuous dynamic recrystallization (DDRX). Rising temperatures and declining strain rates facilitated the emergence and growth of Dynamic recrystallization (DRX) grains. An original JC (O–JC) model and a modified JC (M–JC) model were established. The M–JC model indicated a better prediction than the O–JC model. Still, it was deficient in predicting flow stresses with insufficient coupling effects. Hence, based on the M–JC model, a newly modified JC (NM–JC) model, which further enhances the interaction between strain and strain rate as well as strain and temperature, is proposed. Its projected values can better align with the tested values.

## 1. Introduction

Magnesium alloy is the lowest-density metal structural material with excellent specific strength and stiffness, processing and forming properties, and recyclability [1,2]. It is used extensively in aerospace, automotive, consumer electronics, etc. [3]. Because the dense hexagonal (HCP) lattice structure limits slip systems, the main deformation mechanism at room temperature is twinning. At this time, deformation is hard, and forming quality is tough to ensure [4]. Nevertheless, the deformation mechanism is converted to slips with a lowered critical resolved shear stress, favoring forming at high temperatures [5], so it is usually formed by hot deformation. Thus, studying the deformation behavior of Mg–Gd–Y–Zr–Ag alloy at high temperatures is important.

A hot processing map was developed by dynamic material modeling [6]. The hot processing map separates the hot processing conditions into stable and unstable regions [7]. In the unstable region, microstructural instability of the material may occur, and micro-defects may appear during the deformation process. In the stable region, materials have the best forming performance [8], reflecting the plastic flow behavior of the material, and are useful for selecting and optimizing process conditions [9].

The constitutive model is a numerical model for characterizing the material’s flow behavior under complex conditions and is usually expressed as a math relation among flow stress and related parameters [10,11]. Finite element studies of metal forming processes depend on accurate and reasonable material constitutive models to obtain reliable plastic deformation predictions [12,13]. The constitutive relationship can be obtained by fitting the data acquired by uniaxial compression tests of the material [14]. The present constitutive models are broadly classified into phenomenological, physical basis, and artificial neural network models. The physical basis model contains some physical assumptions based on physical theories such as thermodynamic principles, dislocation motion theory, etc. Still, this model requires more material parameters, which are not easy to obtain, and high experimental accuracy should be guaranteed. The constitutive model established by the neural network isn’t easily implemented in finite element modeling [15,16]. In contrast, phenomenological models contain fewer parameters, which are easy to determine, making them popular in the finite element study of metal-forming processes [17]. Phenomenological models include the JC model.

The JC model considers strain hardening, strain rate hardening, and thermal softening. However, it neglects the coupling effect between the three, which makes it less predictive of flow behavior under certain conditions [18]. Consequently, many researchers have used the JC models to study alloy flow behavior. Hou et al. [19] modified the form of the JC equation’s thermal softening term, making it adaptable to the processing conditions of Mg–Gd–Y alloy in a broad range of temperatures. Feng et al. [20] developed a JC model with cumulative damage that can project the failure behavior of the AZ31 alloy. Abbasi-Bani et al. [21] discovered the JC equation was not well suited for magnesium alloys with significant softening phases. Recently, many scholars [21,22,23,24] improved the predictability of the JC models by accounting for the coupling between parameters. 

In this work, the uniaxial isothermal hot compression test of a high-strength, heat-resistant Mg–Gd–Y–Zr–Ag alloy was performed. Using the test data, a hot processing map was built. The microstructure during thermal deformation was explored using optical microscopy (OM). By combining the flow curves, heat deformation map, and microstructure, the thermal deformation behavior of this alloy was investigated. To characterize the thermal deformation behavior, the NM–JC model was proposed.

## 2. Experimental

The chemical components of the deformed magnesium alloy for the tests are given below (Table 1).

**Figure 1:**
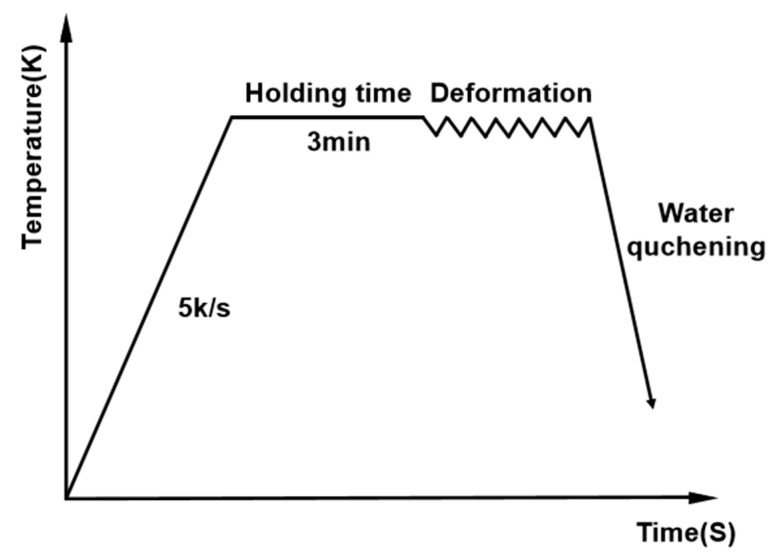
Process flowchart of the isothermal compression process.

A uniaxial isothermal hot compression test was performed on the Gleeble 3180 thermo-mechanical simulator at strain rates of (0.001–1 s^−1^) and temperatures of (623–773 K). Cylindrical specimens with dimensions of Φ10 mm × 15 mm were used. In a thermal compression test, the specimen was heated at 5 K/s and maintained for 3 min after attaining a deformation temperature. It was finally compressed to 60% of the original height at a constant strain rate to collect stress-strain data [25,26], as shown in Figure 1. Four strain rates (0.001 s^−1^, 0.01 s^−1^, 0.1 s^−1^, 1 s^−1^) and four isothermal points (623 K, 673 K, 723 K, 773 K) were designed. 

Following the test, when the sample was allowed to cool in the air, similar to annealing, a significant change in grain size occurred. In addition, magnesium alloys were less susceptible to phase changes during rapid cooling than steel. To preserve the microstructure at the end of this test, the specimen was immersed in water for rapid cooling after the test [26]. Subsequently, the specimens were cut along the axially symmetrical surface, ground, and then etched with a picric acid solution. Then the microstructure was observed and analyzed by optical microscopy (OM). In each condition, repeated at least two tests, the relative error of the two tests does not exceed 10 percent.

## 3. Result and Discussion

### 3.1. Flow Behavior and Microstructural Evolution

**Figure 2:**
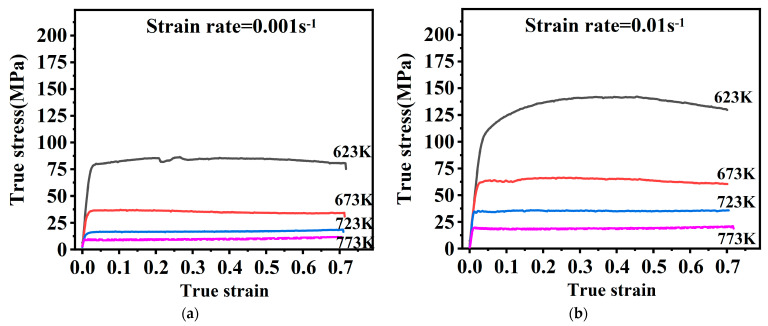

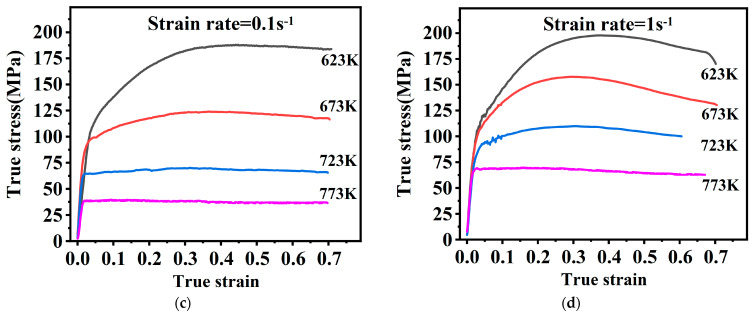
The flow stress curves under different test conditions. (**a**) 0.001s^−1^, (**b**) 0.01s^−1^, (**c**) 0.1s^−1^, (**d**) 1s^−1^.

The flow stress curves gained from uniaxial isothermal compression tests are shown in Figure 2. The stress curves all have a similar regularity. It is a typical phenomenon. With increasing strain, the flow stress rises dramatically, followed by a slow rise, and finally remains stable or slightly decreases without a significant peak.

The flow stress gradually grows in response to strain rate rise and temperature decrease. The flow stresses are mainly dictated through a dynamic interaction of work-hardening and softening mechanisms. Rising temperatures and declining strain rates facilitate the emergence and growth of DRX grains. At this time, the softening mechanism is strengthened, and the stress drops. In addition, in Figure 2d, at 723 K, there are small serrations in the yielding stage. This is related to the dynamic strain aging effect resulting from interactions of the alloy solute atoms with dislocation motion. A similar observation was reported by Zhang [27].

To further investigate the flow behavior, a hot processing map was drawn using the dissipative structure theory. It reflects the plastic deformation capacity and the power consumption ratio of energy used to change the microstructure in each process condition. The power consumption rate η is related to the strain-rate-sensitivity index *m*, represented in Equation (1) [28].

(1) $$\eta =\frac{2m}{m+1}$$

Combined with the instability criterion presented in Equation (2) [28], it can determine whether the material is stable under the corresponding deformation conditions.

(2) $$\xi =\frac{\partial \ln(\frac{m}{m+1})}{\partial \ln\overset{•}{\varepsilon }}<0$$

**Figure 3:**
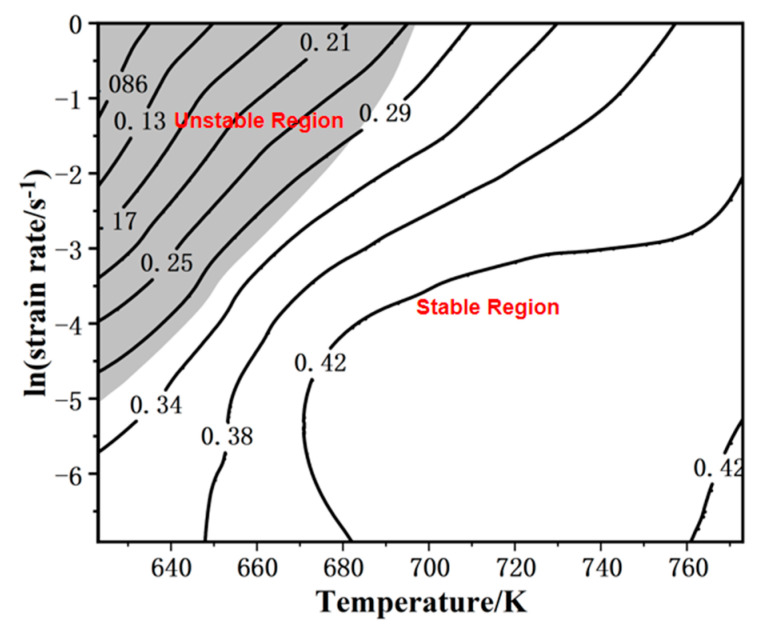
The hot processing map for Mg–Gd–Y–Zr–Ag alloy.

The energy dissipation rate map and instability map are available by calculating the test data with Equations (1) and (2). The hot processing map of the magnesium alloy can be drawn by combining these two maps (Figure 3).

**Figure 4:**
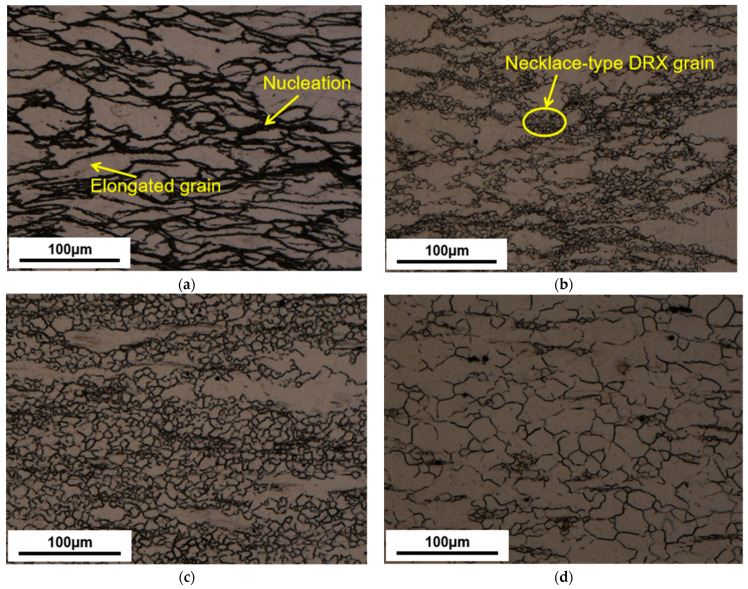
The samples’ microstructure with different temperatures at 0.01 s^−1^. (**a**) 623 K, (**b**) 673 K, (**c**) 723 K, (**d**) 773 K.

**Figure 5:**
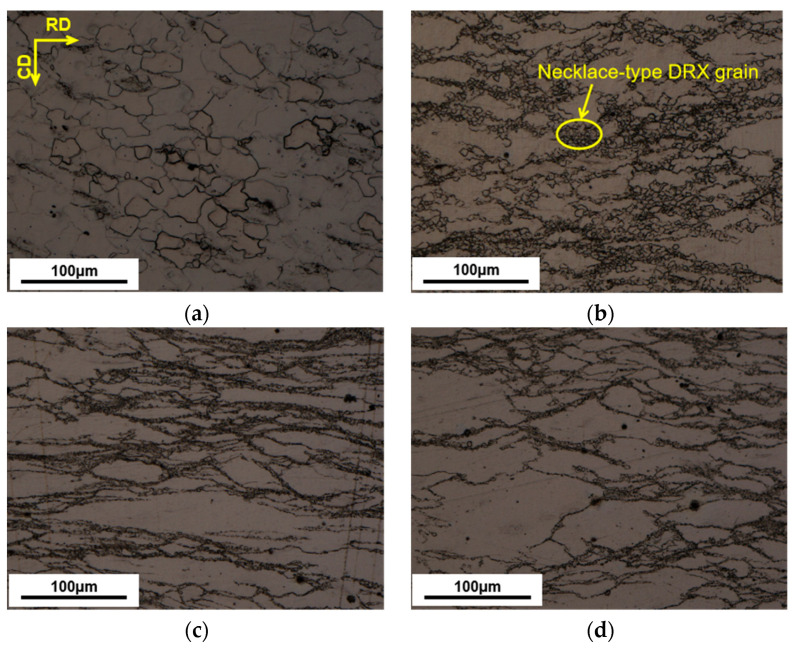
The samples’ microstructure with different strain rates at 673 K. (**a**) 0.001 s^−1^, (**b**) 0.01 s^−1^, (**c**) 0.1 s^−1^, (**d**) 1 s^−1^.

Figure 3 demonstrates the deformation conditions (673–773 K and 0.001–1 s^−1^) in the stable region when the power consumption rate is 31–42%. This is the dynamic recrystallization (DRX) active region. In Figure 4, significant DRX grains appear when the temperature exceeds 673 K. Deformation conditions in this region are conducive to plastic forming. The critical state is at 373 K–1 s^−1^, when ξ is almost 0. It is also verified in Figure 5d. In this case, there are fewer dynamically recrystallized grains, and almost no DRX grains are distributed near some grain boundaries.

In the unstable region, as in Figure 4a, obvious DRX grains fail to develop because of a lower forming temperature. Deformation conditions in this region are unfavorable for plastic forming. Under this situation, micro-defects may occur [28]. In Figure 2a, at 623 K, there is a hump, which may result from it being in an unstable region where the work-hardening and softening mechanisms are in an unstable equilibrium [29]. The temperature should be kept at least 673 K for this magnesium alloy to ensure the deformation quality.

Figure 4a–d demonstrates the sample’s microstructure with deformation temperatures of 623–773 K at 0.01 s^−1^. Figure 4a indicates primitive grains are stretched perpendicular at the deformation orientation (CD). There is a clear nucleation near the original grain boundaries for temperatures at 623 K. As seen in Figure 4b, the nucleation gradually grows and presents a typical necklace-type DRX grain along the primitive grain boundary when the temperature reaches 673 K. As seen in Figure 4c, the DRX grains almost replaced the original grains when the temperature reached 723 K. As seen in Figure 4d, when the temperature reached 773 K, the DRX grains enlarged further until the primitive grains were replaced entirely.

**Figure 6:**
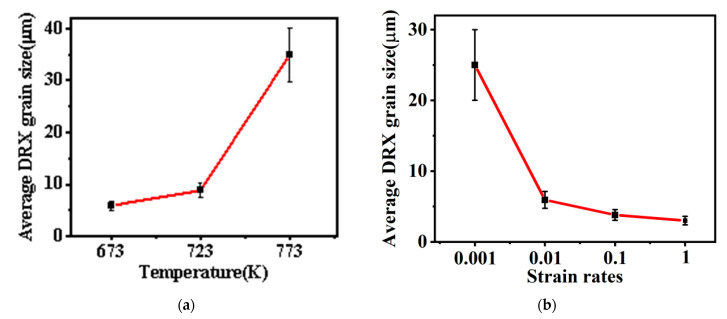
Average DRX grain size of the tested magnesium alloy samples deformed (**a**) at different temperatures and (**b**) at different strain rates.

Figure 6a demonstrates the average DRX grain size under different temperatures. Figure 4 and Figure 6a show that, as the temperature rises, the size and the volume proportion of DRX grains will increase and finally homogenize to replace the primitive grain. This is attributed to the high deformation temperature increasing the driving force of dislocations. At the same time, the driving force promotes the grain boundary migration motion and occurrence of DRX. 

Figure 5a–d demonstrates the sample’s microstructure at 673 K and 0.001–1 s^−1^. Figure 5a shows that the earliest grains are almost replaced by larger DRX grains at 0.001 s^−1^. As seen from Figure 5b–d, necklace-type DRX grains are distributed around the primitive grain boundaries. The volume proportion of DRX grains declines significantly as its strain rate increases. As seen in Figure 5 and Figure 6b, with a decrease in strain rate, DRX grains will be larger, and the volume proportion will increase. This is owing to the slower deformation of the material, which gives sufficient time for DRX nucleation and grain growth.

Dislocation climb and cross-slip are limited by magnesium alloys’ lower SFE, which results in a lower dynamic rate of recovery and higher dislocation density, which facilitates the occurrence of dynamic recrystallization [30,31]. Figure 4 and Figure 5 show that one of the major deformation mechanisms at high temperatures is DRX. The nucleation of this DRX occurs along the primitive grain boundaries with distinct nucleation and growth phases, characteristic of discontinuous dynamic recrystallization (DDRX) [32]. In combined Figure 2, overall, there are no significant peaks in the rising phase of flow stress, indicating that the main softening mechanism is DDRX at high temperatures.

### 3.2. Johnson-Cook Model

The original JC model (O–JC) is a structurally simple function expressed as a math relation among flow stress and deformation parameters, represented in Equation (3) [28].

(3) $$\sigma =(A+B\varepsilon^{n})[1+C\ln(\overset{•}{\varepsilon }/\overset{•}{\varepsilon_{0}})][1-{\left(\frac{T-T_{r}}{T_{m}-T_{r}}\right)}^{m}]$$

σ, ε, $\overset{•}{\varepsilon }$, T is the flow stress, strain, strain rate, and temperature, respectively. $T_{r}$ and $\overset{•}{\varepsilon_{0}}$ refer to the reference values of 623 K and 0.001 s^−1^ for strain and temperature, respectively. A denotes yield stress under reference conditions (65.23 MPa). $T_{m}$ is melting point temperature (920 K). B, C, n, m is the material constant. In the reference condition, the model is translated into Equation (4) [28].

(4) ln(σ−A)=lnB+nlnε

According to a relationship of ln(σ−A) and lnε, then constants B and n will be determined at 22.291 and 0.082, respectively. Equation (3) is translated into Equation (5) [33] at 623 K.

(5) $$\frac{\sigma }{A+B\varepsilon^{n}}-1=C\ln(\overset{•}{\varepsilon }/\overset{•}{\varepsilon_{0}})$$

According to a relationship of $\sigma /(A+B\varepsilon^{n})-1$ and $\ln(\overset{•}{\varepsilon }/{\overset{•}{\varepsilon }}_{0})$, the constant C can be computed as 0.117. Equation (3) is translated into Equation (6) [34] at 0.001 s^−1^.

(6) $$\ln(1-\frac{\sigma }{A+B\varepsilon^{n}})=m\ln(\frac{T-T_{r}}{T_{m}-T_{r}})$$

According to the relationship of $\ln\;[1-\sigma /(A+B\varepsilon^{n})]$ and $\ln[(T-T_{r})/(T_{m}-T_{r})]$, the constant m can be computed as 0.264. So far, the O–JC model is shown in the following equation:

(7) $$\sigma =(65.23+22.291\varepsilon^{0.082})[1+0.117\ln(\overset{•}{\varepsilon }/\overset{•}{\varepsilon_{0}})][1-{\left(\frac{T-T_{r}}{T_{m}-T_{r}}\right)}^{0.264}]$$

**Figure 7:**
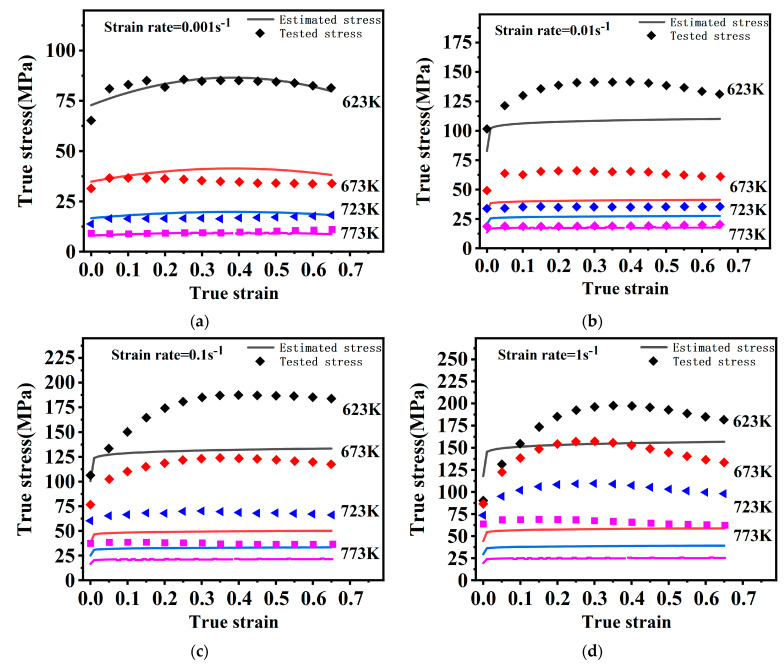
Tested stress and estimated stress by the O–JC under different conditions. (**a**) 0.001 s^−1^, (**b**) 0.01 s^−1^, (**c**) 0.1 s^−1^, (**d**) 1 s^−1^.

The tested stress and estimated stress by the O–JC are presented in Figure 7. Obviously, the original JC model neglects the coupling effect between the three influencing factors, which leads to worse predictability. The O–JC model predicts poorly under other deformation conditions except for the reference strain rate. The prediction gets worse with higher strain rates and temperatures.

### 3.3. Modified Johnson-Cook Model

With higher strain rates and temperatures, the O–JC model must be improved in predicting flow stresses. Lin et al. [35] took the interaction between temperature and strain rate into consideration to establish a modified JC model (M–JC) with enhanced coupling effects, as shown in Equation (8).

(8) $$\sigma =(A_{1}+B_{1}\varepsilon +B_{2}\varepsilon^{2})[1+C\ln(\overset{•}{\varepsilon }/\overset{•}{\varepsilon_{0}})]\exp\{[\lambda_{1}+\lambda_{2}\ln(\overset{•}{\varepsilon }/\overset{•}{\varepsilon_{0}})](T-T_{r})\}$$

where $A_{1}$, $B_{1}$, $B_{2}$, C, $\lambda_{1}$, $\lambda_{2}$ is the material constant. The rest parameters match the O–JC model. Equation (8) is transformed into Equation (9) [35] at 623 K and 0.001 s^−1^.

(9) $$\sigma =A_{1}+B_{1}\varepsilon +B_{2}\varepsilon^{2}$$

The experimental data at the reference condition was subjected to quadratic polynomial fitting to obtain $A_{1}$, $B_{1}$, $B_{2}$.The parameters $A_{1}$, $B_{1}$, $B_{2}$ are computed as 72.849 Mpa, 71.755 Mpa, and −93.911 Mpa, respectively. At 623 K, Equation (8) is transformed into Equation (10) [35].

(10) $$\frac{\sigma }{A_{1}+B_{1}\varepsilon +B_{2}\varepsilon^{2}}-1=C\ln(\overset{•}{\varepsilon }/\overset{•}{\varepsilon_{0}})$$

According to the relationship of $\sigma /(A_{1}+B_{1}\varepsilon +B_{2}\varepsilon^{2})-1$ and $\ln(\overset{•}{\varepsilon }/\overset{•}{\varepsilon_{0}})$, the constant C can be computed as 0.142. To solve for $\lambda_{1}$ and $\lambda_{2}$, a parameter k is introduced. Equation (8) is transformed into Equation (11) [35] at 0.001 s^−1^.

(11) $$\{\begin{array}{c} \ln\left[\frac{\sigma }{\left(A_{1}+B_{1}\varepsilon +B_{2}\varepsilon^{2})[1+Cln(\overset{•}{\varepsilon }/{\overset{•}{\varepsilon }}_{0})\right]}\right]=k\left(T-T_{r}\right)\\ k=\lambda_{1}+\lambda_{2}ln(\overset{•}{\varepsilon }/{\overset{•}{\varepsilon }}_{0}) \end{array}$$

**Figure 8:**
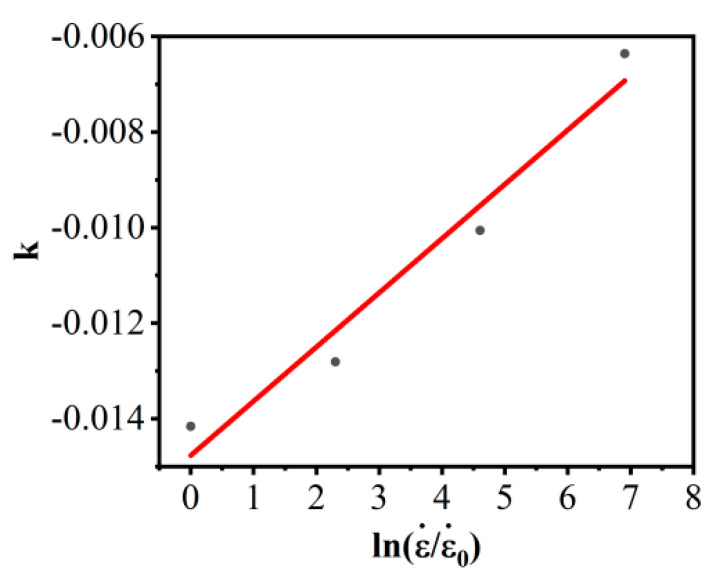
The correlation of k and $\ln(\overset{•}{\varepsilon }/{\overset{•}{\varepsilon }}_{0})$ in M–JC.

According to the relationship of $\ln\{\sigma /[(A_{1}+B_{1}\varepsilon +B_{2}\varepsilon^{2})[1+Cln(\overset{•}{\varepsilon }/{\overset{•}{\varepsilon }}_{0})]]\}$ and $T-T_{r}$, the corresponding parameters k can be solved. When the strain rate is 0.001 s^−1^, 0.01 s^−1^, 0.1 s^−1^, and 1 s^−1^, the corresponding parameters *k* are −0.01416, −0.01281, −0.01006, and −0.00636, respectively. The correlation of *k* and $\ln(\overset{•}{\varepsilon }/{\overset{•}{\varepsilon }}_{0})$ is shown in Figure 8. Then $\lambda_{1}$, $\lambda_{2}$ can be computed as −0.0148 and 0.00114, respectively.

The M–JC model is shown in the following equation:

(12) $$\sigma =\left(72.849+71.755\varepsilon -93.911\varepsilon^{2}\right)\left(1+0.142\ln(\overset{•}{\varepsilon }/\overset{•}{\varepsilon_{0}})\right)\exp\left[\left(-0.01477+0.00114\ln(\overset{•}{\varepsilon }/\overset{•}{\varepsilon_{0}})\right)\left(T-T_{r}\right)\right]$$

**Figure 9:**
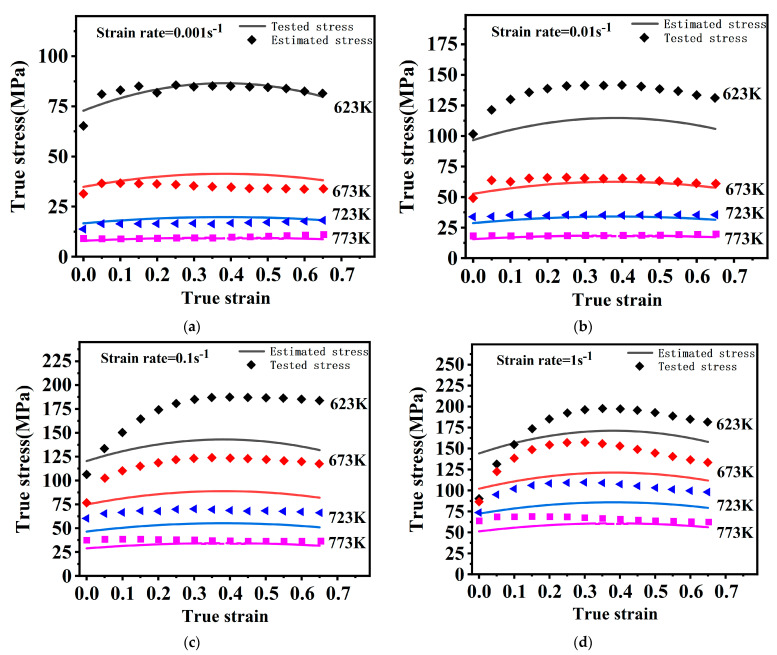
Tested stress and estimated stress by M–JC under different conditions. (**a**) 0.001 s^−1^, (**b**) 0.01 s^−1^, (**c**) 0.1 s^−1^, (**d**) 1 s^−1^.

The tested stress and estimated stress by M–JC are presented in Figure 9, which indicates a better prediction than the O–JC model. At 0.001 s^−1^ and 0.01 s^−1^, overall predictions are best. However, the model’s prediction deteriorates with either decreasing temperature or growing strain rates.

### 3.4. Newly Modified Johnson-Cook Model

Obviously, the M–JC is still deficient in predicting flow stresses because they do not sufficiently consider the interaction between strain, strain rate, and temperature. Hence, based on the M–JC model, a newly modified JC (NM–JC) model, which further enhances the interaction between strain and strain rate as well as the interaction between strain and temperature, is proposed, represented in Equation (13).

(13) $$\{\begin{array}{c} \sigma =(A+B\varepsilon^{n})(1+C\ln(\overset{•}{\varepsilon }/\overset{•}{\varepsilon_{0}}))\exp\{[\lambda_{1}+\lambda_{2}\ln(\overset{•}{\varepsilon }/\overset{•}{\varepsilon_{0}})+\lambda_{3}{\left(\ln(\overset{•}{\varepsilon }/\overset{•}{\varepsilon_{0}})\right)}^{2}]\left(T-T_{r}\right)\}\\ \begin{array}{l} B=B_{1}\{1+B_{2}\ln(T/T_{0})+B_{3}{\left[\ln(T/T_{0})\right]}^{2}\}\\ C=C_{1}+C_{2}\varepsilon +C_{3}\varepsilon^{2} \end{array} \end{array}$$

where $B_{1}$, $B_{2}$, $B_{3}$, $C_{1}$, $C_{2}$, $C_{3}$, $\lambda_{1}$, $\lambda_{2}$, $\lambda_{3}$ is the material constant. Other parameters are consistent with the O–JC model. At the reference condition, consider B as a constant-type parameter. Parameter *n* is solved similarly to the O–JC model. Parameter *n* is 0.082. Parameter B is temporarily considered to be the constant 22.291. At 623 K, Equation (13) is transformed into Equation (14).

(14) $$(\frac{\sigma }{A+B\varepsilon^{n}}-1)/\ln(\overset{•}{\varepsilon }/\overset{•}{\varepsilon_{0}})=C_{1}+C_{2}\varepsilon +C_{3}\varepsilon^{2}$$

The material constants $C_{1}$, $C_{2}$, $C_{3}$ were obtained by fitting a quadratic polynomial to $\left[\sigma /(A+B\varepsilon^{n})-1]/\ln(\overset{•}{\varepsilon }/\overset{•}{\varepsilon_{0}}\right)$ and ε. Constants $C_{1}$, $C_{2}$, $C_{3}$ are 0.114, 0.678, and −0.850, respectively. An intermediate parameter *k* is introduced to solve the parameters $\lambda_{1}$, $\lambda_{2}$, $\lambda_{3}$.Equation (13) is translated into Equation (15) at 0.001 s^−1^.

(15) $$\{\begin{array}{c} \ln\left[\frac{\sigma }{\left(A+B\varepsilon^{n})[1+C\ln(\overset{•}{\varepsilon }/\overset{•}{\varepsilon_{0}})\right]}\right]=k\left(T-T_{r}\right)\\ k=\lambda_{1}+\lambda_{2}\ln(\overset{•}{\varepsilon }/\overset{•}{\varepsilon_{0}})+\lambda_{3}{\left[\ln(\overset{•}{\varepsilon }/\overset{•}{\varepsilon_{0}})\right]}^{2} \end{array}$$

**Figure 10:**
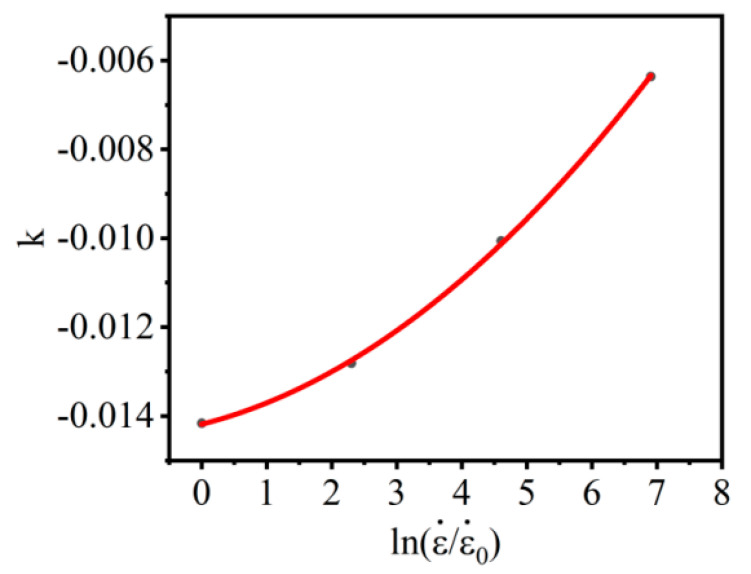
The correlation of k and in NM–JC.

According to the relationship of $\ln\{\sigma /[(A+B\varepsilon^{n})[1+C\ln(\overset{•}{\varepsilon }/{\overset{•}{\varepsilon }}_{0})]]\}$ and $T-T_{r}$, the corresponding parameters k can be solved. When the strain rate is 0.001 s^−1^, 0.01 s^−1^, 0.1 s^−1^, and 1 s^−1^, the values of the corresponding parameters k are −0.0142, −0.0128, −0.0101, 0.00037, respectively. The correlation of k and $\ln(\overset{•}{\varepsilon }/{\overset{•}{\varepsilon }}_{0})$ is shown in Figure 10. Then $\lambda_{1}$, $\lambda_{2}$, $\lambda_{3}$ can be computed as −0.0142, 0.000307, 0.000111, respectively.

At this time, all parameters except for parameters $B_{1}$, $B_{2}$, $B_{3}$ are known. The NM–JC model can be formalized as.

(16) $$\{\begin{array}{c} \sigma /Q-A=B_{1}+B_{1}B_{2}\ln(T/T_{0})+B_{1}B_{3}[\ln(T/T_{0})]\\ Q=1+C\ln(\overset{•}{\varepsilon }/\overset{•}{\varepsilon_{0}})\exp\{(\lambda_{1}+\lambda_{2}\ln(\overset{•}{\varepsilon }/\overset{•}{\varepsilon_{0}})+\lambda_{3}{\left[\ln(\overset{•}{\varepsilon }/\overset{•}{\varepsilon_{0}})\right]}^{2})(T-T_{r})\} \end{array}$$

According to the relationship of σ/Q−A and $\ln(T/T_{0})$, the material parameters $B_{1}$, $B_{2}$, $B_{3}$ are solved as 20.628, −1.023, 2.767, respectively. The NM–JC model is shown in the following equation:

(17) $$\{\begin{array}{c} \sigma =(65.23+B\varepsilon^{n})[1+C\ln(\overset{•}{\varepsilon }/\overset{•}{\varepsilon_{0}})]\exp\{[-0.0142+0.000307\ln(\overset{•}{\varepsilon }/\overset{•}{\varepsilon_{0}})+0.000111{\left(\ln(\overset{•}{\varepsilon }/\overset{•}{\varepsilon_{0}})\right)}^{2}]\left(T-T_{r}\right)\}\\ \begin{array}{l} B=20.628\{1-1.023\ln(T/T_{0})+2.767{\left[\ln(T/T_{0})\right]}^{2}\}\\ C=0.114+0.678\varepsilon -0.85\varepsilon^{2} \end{array} \end{array}$$

**Figure 11:**
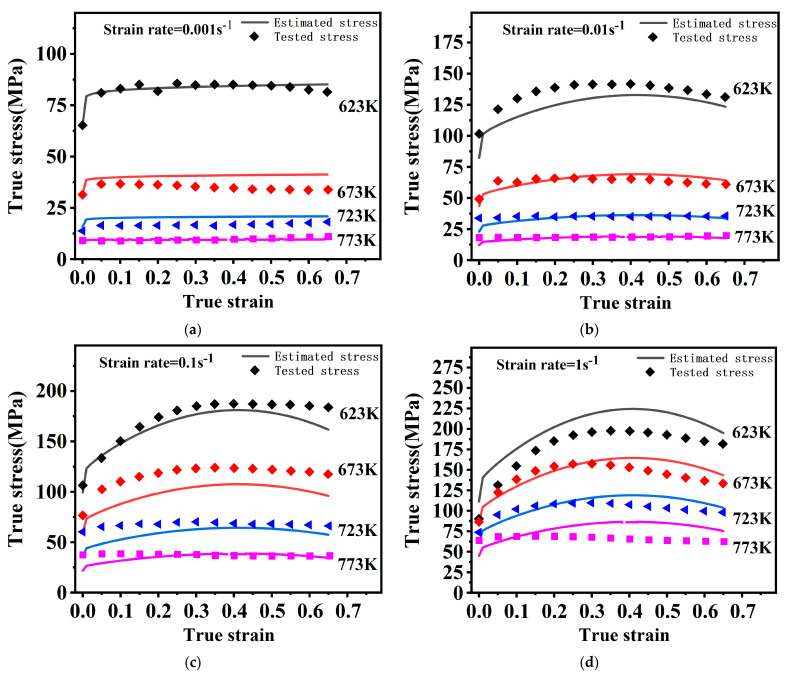
Tested stress and estimated stress by the NM–JC under different conditions. (**a**) 0.001 s^−1^, (**b**) 0.01 s^−1^, (**c**) 0.1 s^−1^, (**d**) 1 s^−1^.

The tested stress and estimated stress by the NM–JC model are presented in Figure 11. The NM–JC model involves strengthened coupling effects that can effectively predict flow behaviors at 623–773 K and 0.001–1 s^−1^.

## 4. Analysis of the NM–JC Model Predictability

To evaluate the predictiveness of the constitutive models established in this study. The R, RE, and AARM were utilized.

(18) $$\mathrm{Correlation}\;\mathrm{coefficient}(R)=\frac{\sum_{i=1}^{n}\left(T_{i}-\bar{T})(E_{i}-\bar{E}\right)}{\sqrt{\sum_{i=1}^{n}{\left(T_{i}-\bar{T}\right)}^{2}\sum_{i=1}^{n}{\left(E_{i}-\bar{E}\right)}^{2}}}$$

(19) $$\mathrm{Relative}\;\mathrm{error}(\mathrm{RE})=\left|\frac{T_{i}-E_{i}}{T_{i}}\right|\times 100\%$$

(20) $$\mathrm{Average}\;\mathrm{relative}\;\mathrm{error}(\mathrm{AARE})=\frac{1}{n}\sum_{i=1}^{n}\left|\frac{T_{i}-E_{i}}{T_{i}}\right|\times 100\%$$

T, E are the tested and estimated date, respectively. *n* is the sample size. $\overset{\_\_}{T}$, $\overset{\_\_}{E}$ are the average test and estimated date, respectively.

**Figure 12:**
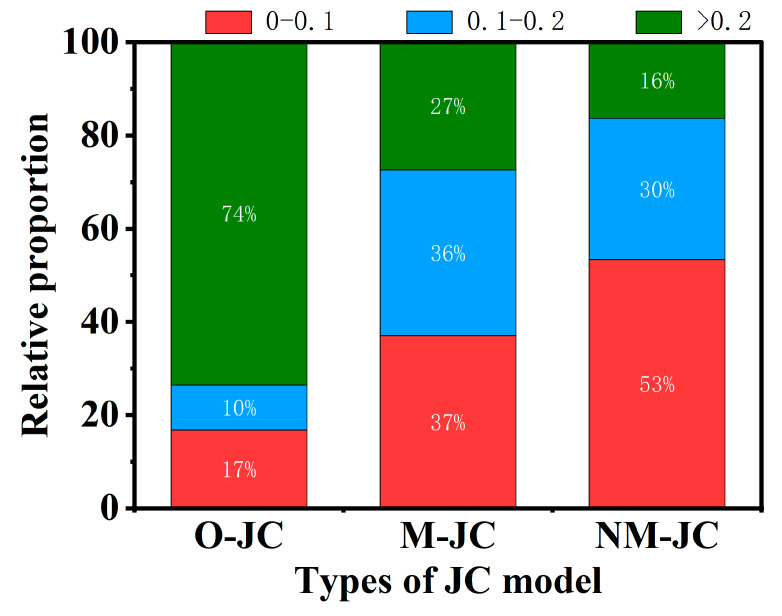
The relative proportion of the range of RE values.

This study involved 224 stress-strain sample points in calculating the constitutive model. Figure 12 shows the relative proportion of the range of RE values for the three different JC model prediction values. Compared with the O–JC model and M–JC model, the RE values for NM–JC model prediction values in the range of 0–0.1 increased significantly. In contrast, the RE values greater than 0.2 decreased significantly. The RE values for NM–JC model prediction values in the range of 0–0.1 accounted for 53%, in the range of 0.1–0.2 accounted for 30%, and only 16% were greater than 0.2.

**Figure 13:**
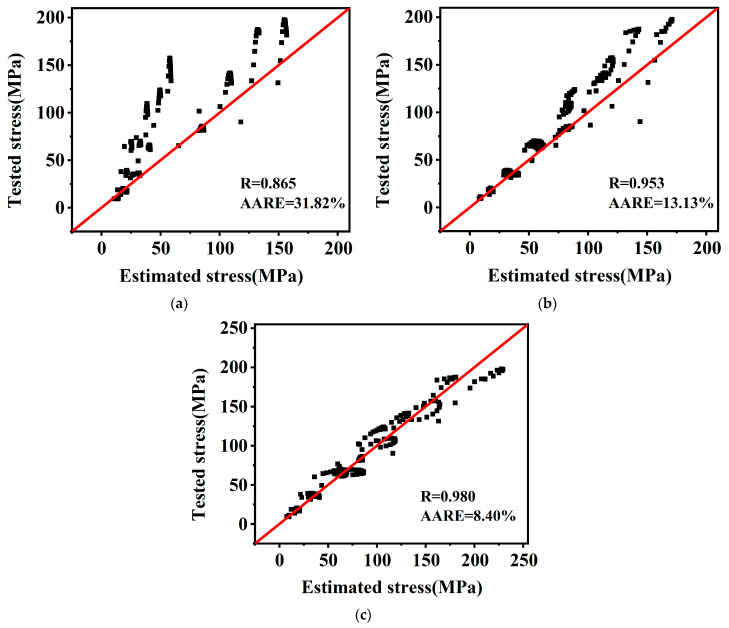
Correlation between tested and estimated flow stresses: (**a**) O–JC model; (**b**) N–JC model; (**c**) NM–JC model.

Figure 13 shows the R of the O–JC model, the M–JC model, and the NM–JC model are 0.865, 0.953, 0.980, respectively, and the average relative error (AARM) is 31.82%, 13.13%, and 8.40%, respectively. The NM–JC model has a better correction than the two previous models. Better predictability of flow stresses for this material is provided by the NM–JC model.

## 5. Conclusions

The thermal deformation behavior of Mg–Gd–Y–Zr–Ag alloy at 623–773 K and 0.001–1 s^−1^ was researched through uniaxial isothermal compression tests. Some specific conclusions are as follows:

(1) Strain, strain rate, and temperature directly impact the deformation behavior. The flow stress increases with increased strain rates and decreased temperature, while it first increases and finally remains stable with increased strain;
(2) The hot processing map reveals the deformation temperature should be kept at 673–773 K for this alloy when the power consumption rate is 31–42%;
(3) During the high temperature deformation process, the main softening mechanism is DDRX. The DRX grains will be larger, and the volume proportion of DRX grains will also increase in response to the temperature rise or strain rate decrease;
(4) The NM–JC model more perfectly predicts the thermal deformation behavior of this magnesium alloy compared with the O–JC and M–JC models. The R and AARE of the flow stresses predicted by NM–JC reached 0.980 and 8.4%, respectively.

## Figures and Tables

**Table 1:** Components of the alloy (wt.%).

Gd	Y	Zr	Ag	Mg
8.0~9.6	1.8~3.2	0.3~0.7	0.02~0.50	Bal

## Data Availability

Not applicable.
